# Vestiges of the Natural History of Creation

**Published:** 1845-09

**Authors:** 


Art. V.—*Vestiges of the Natural History of Creation.* Second edition, from the third London edition, greatly amended by the author; and an Introduction, by Rev. George B. Cheever, D. D. New York: Wiley & Putnam. 1845. 12mo. pp. 280.

The author of this singular volume is not generally known. We have seen it stated that he is a member of Parliament, and of the name of Vyvyan. His work has been very extensively read, as is shown by the fact that in a little more than a year it has passed through three editions in London, and two in this country. It is well calculated to excite interest, and will probably continue for some time yet to attract a good deal of attention, for it has that element which the French lady felt to be wanting in the glass of water when she exclaimed, as she raised the pure antediluvian beverage to her lips, "*what a pity it is not a sin!*" It has the charm of "stolen waters;" it comes in at least a questionable shape, if it is not clearly contraband. Thus the Medico-Chirurgical Review, in reference to one of the hypotheses of the author, says, "it is three or four centuries in advance of the times, and will be stigmatised by the modern saints as downright atheism."

From this single observation the reader will be able to divine the character of this production. Doctor Cheever has deemed it well to introduce it to American readers by some very spirited and eloquent objections, in which the doctrines of the work are handled with severity. He finds in them all to alarm the "saint," that the reviewer aforesaid thinks is so far in advance of "the ignorant present times." We incline to believe that Dr. Cheever has pushed some of his strictures too far, and has betrayed some of that bigotry which the sneering critic considers an attribute of "the modern saints;" but the zeal, which may be carried too far, is displayed in a good cause, and the speculations assailed are certainly obnoxious to the severest criticism. It is true, that they have been somewhat modified in succeeding editions of the work, and that the author has even gone so far in deference to public opinion as to withdraw some of the revolting conclusions which he set forth at first as derivable from his doctrines; still, as his commentator holds, the system is answerable for the results which flow from it, and must be judged by the conclusions to which it leads, whether the author carries them out or not.

We propose looking a little into this system, and will say at the beginning, that it rests upon a curious assemblage of facts, which render the volume worthy of an attentive perusal. The author is not only a classical and elegant writer, but evidently a man who has made himself extensively acquainted with science, and he brings from physiology, comparative anatomy, chemistry, and geology a multitude of illustrations to exemplify and support his hypothesis. His facts come thus, to be not only varied and curious, but in the truest sense of the word highly interesting, and it is only with his deductions from the facts, that his reviewers have found cause of quarrel. The hypothesis, which it is the design of the volume to unfold and defend, is, that the creation of the earth and all its inhabitants is the result of a *law of development.* The earth, he supposes, with La Place and others, existed first as a vapor, which gradually cooled down, and assumed the solid form. After a time, by a law pre-established, the lowest races of animals were created, and by development, one of these races was brought out of another—birds out of fishes, and mammals out of birds, until the highest of these, the monkeys, gave birth to the human race.

This is the hypothesis, and at this point we fear many readers will stop, resolved to have nothing more to do with a book reviving so ridiculous a doctrine. But any one who takes this resolution will do himself injustice, for absurd as this notion is, the work is not one to be despised. Let us pursue the history of creation as it is therein given.

The primary condition of matter, according to this hypothesis, was that of a diffused mass, portions of which in time were agglomerated into suns, which by a centrifugal force threw off planets; the earth was thrown off from the sun before its sister Venus, and its younger brother Mercury, and after Mars, Jupiter, Saturn, and Uranus; the nebulous matter of which it consisted by the opposite law of attraction collected around nuclei, and gradually contracted by cooling to its present dimensions. For a long time the earth was under the dominion of fire, and its primary rocks are hence crystalline, without any traces of animal or vegetable existence. But the progressive development went on; the primary rocks were upheaved by volcanoes, and worn by the atmosphere and water, and gave birth to the stratified rocks. In these we find the earliest vestiges of living creation. Up to the deposit of mineral matter from the seas, the geologist has no decisive proof that any vital being existed upon the earth. Still, as our author remarks, there is "difficulty with regard to the very first chapter of Fauna's story," and there is room to suspect that even the early ocean was peopled by some simple forms of being, which have "left no memorial of their slight gelatinous forms" in the rocks which formed around them. The heat to which these rocks have been subjected would account for the obliteration of every trace of such animals, as well as of the fragile plants which may have flourished in the earliest seas.

In the mica state and grauwacké slate systems indisputable vestiges of animal life are seen, in the fossil remains of polypi, encrinites, crustacea and conchifera, the two first belonging to the humblest of Cuvier's four divisions of the animal kingdom, the radiata, the other two to the next divisions, the articulata and mollusca.

"In common, though not very precise language, they are corals and shell-fish. Nothing uncommon or surprising is to be observed in their forms; but it is remarkable that, though they can readily be referred to existing *orders*, the species and even genera to which they belonged are no longer found on earth; nay, almost the whole had become extinct before the next group of strata was formed. Such changes of species we shall find to be of frequent occurrence throughout the subsequent ages. Of the crustacea of the system, the most remarkable forms are trilobites—animals which continued to flourish to a great variety of species throughout several of the subsequent rock-formations, but which are now only faintly represented in a few obscure species. Some curious inferences have been made by Dr. Buckland from the prominent facet-covered eyes with which this creature was furnished, indicating that the sea in which it lived was a clear medium, as existing seas generally are, and that light was the same in character in those inconceivably remote ages as it is now.

"Ascending to the next group of rocks, we find the traces of life become more abundant, the number of species extended, and important additions made in vestiges of fuci, or sea plants, and of fishes." pp. 42, 43.

The temperature of the earth in those primeval times seems to have been uniform over its entire surface, owing as Brogniart conjectures, to the internal heat, which was so great as to "overpower the ordinary meteorological influences;" and with this uniform tropical climate there appears to have been a general identity of organic life over the globe, remains of the same species of animals being found in England, Germany, Norway, Russia, and the Valley ot the Mississippi, seven thousand miles asunder. Species, in the same low class of animals, are now so much more limited, that even the Red Sea and the Mediterranean give different polypiaria, zoophytes, and shell-fish.

"We advance to a new chapter in this marvellons history —the era of the *old red sand-stone system.*" The forms of life discovered in the former series of rocks are continued in this, but to these are added numerous fishes. Prof. Agassiz has made out twenty genera, and sixty species, which lived in the seas from which those strata were deposited. They were fishes of a low order of vitality, and are but slenderly represented by any of the inhabitants of existing seas. They were, like the animals of the previous era, widely diffused, their remains being found in "the corresponding strata of distant parts of the earth." With these aquatic beings marine plants appear in greater abundance, and the vegetation seems to have been less changed in successive eras than the animals, which is accounted for by the gradual infiltration of saline matters into the waters of the sea. These being unifriendly to certain species of aquatic animals, but not affecting marine plants, would bring about frequent changes in the former, and leave the latter undisturbed through a succession of ages.

As yet there were no land animals or plants, and for the very sufficient reason, that no dry land as yet existed. But internal forces at length upheaved the bottoms of the seas, and dry land appeared, first at one point, then at another, and in America before Europe, for stratification affords criteria by which geologists can determine with accuracy the ages of mountains, and these proclaim our continent to be the older of the two. Fresh water accompanied the appearance of land, and the earth was now a fit theatre for other classes of beings. Do we find traces of them in the strata of that period? We do. Vegetation flourished in the utmost profusion, and its decay has left the beds of coal which make the mineral wealth of many nations. It has been called the *carbonigenous* era. The plants were rude and unlovely; no flowers adorned them, and they bore no fruit, but they were luxuriant beyond what the most fertile spots on earth can now show; and it will be remembered that there were then no men or animals on earth to be affected by their barrenness. The office they performed was one of the highest importance; they seem to have been commissioned to remove from the atmosphere the excess of carbonic acid, thus adapting it to the respiration of the more perfect animals, and having fulfilled their destiny were submerged, and carbonized, and piled up in mountain masses for the benefit of the human race. The animal remains of this era are not numerous compared with those that go before, or those that follow.

"The mountain lime-stone, indeed, deposited at the commencement of it, abounds unusually in polypiaria and crinoidea; but when we ascend to the coal-beds themselves the case is altered, and these marine remains altogether disappear. We have then only a limited variety of shell mollusks, with fragments of a few species of fishes, and these are rarely or never found in the coal seams, but in the shales alternating with them. At this time the sauroids, a family of the ganoid fishes are considered as at their apogee, or point of greatest abundance; a fact of some importance, seeing that in teeth, bones, and scales they make an advance to the lizard character, a type of a higher order of animals which we are soon to see entering upon the stage." p. 66.

The termination of the carboniferous formation is marked by indications of volcanic violence, "which some geologists have considered to denote the close of one system of things, and the beginning of another." The earth presented now large tracts of dry land, and the atmosphere had become fit for animals of a higher organization. Terrestial zoology commences at this point, namely with the *new red sand-stone system.* The animals were lizards, one grade above fishes; their imperfect respiratory organs being adapted to an atmosphere not yet quite suitable for birds or mammalia. Some of them were of gigantic size; the *ichthyosaurus*, for example, was as long as a young whale, and was fitted for living in the water, though breathing the atmosphere. The *plesiosaurus* was of similar bulk; in shape it resembled the turtle, but it had a long serpent-like neck, terminating in a head fitted to capture its prey at a considerable distance. These two animals are supposed to have lived in the shallow borders of the seas of that era, devouring immense numbers of the finny tribes. They had as companions the *megalosaurus*, an enormous lizard, also carnivorous and inhabiting the land, and immense crocodiles which fed on plants. And not only these, but birds also of gigantic stature, for the proof has lately been brought to light, in the shape of foot-prints on the new red sand-stone of the Connecticut, that thus early animals so advanced on the scale of being had begun to exist. But we are to bear in mind that geologists divide time by eras, which may embrace many thousands of years, and that while the lizards flourished at the beginning of one of these, birds did not make their appearance until towards the close of it, when the air had undergone the improvement necessary to their existence. It is only in this way, it appears to us, that we get clear of the absurdity of supposing birds to have flourished under a state of things in which reptiles alone could have lived, unless, which is probably the fact, we conclude that these primeval creatures were not birds of flight, and did not possess the highly developed respiratory organization which we find in the present tenants of the atmosphere. So the fact is, however, that *struthious* birds ,resembling the ostrich, but larger, have left their footsteps upon rocks cf the same system as those which contain the remains of the extinct tribes of lizards. None of their fossils have been found on this continent; but there is no longer a doubt that these foot-prints are the real tracks of birds; and, indeed, since their discovery, Professor Owen, of London, has received from New Zealand skeletons of several species of birds, now extinct, which belonged to the same ancient family.

The next era is that of insects, and the mammalia. In the Stones-field slate there have been found several specimens of the lower jaw-bone of an insectivorous quadruped of the marsupial family. It is an interesting circumstance, as the author of the Vestiges remarks, that the first mammifers found should have belonged to that family, when the place of that order in the scale of creation is considered. It was low, as will be seen from the following description:

"In the imperfect structure of their brain, deficient in the organs connecting the two hemispheres—and in the mode of gestation, which is only in part uterine—this family is clearly a link between the oviparous vertebratæ (birds, reptiles, and fishes) and the higher mammifers. This is further established by their possessing a faint development of two canals passing from near the anus to the external surface of the viscera, which are fully possessed in reptiles and fishes, for the purpose of supplying ærated water to the blood circulating in particular vessels, but which are unneeded by mammifers."

In the next period the mammals become abundant. All over the chalk-beds where deposits of clay, lime-stone, and marl have taken place, constituting *tertiary* formations, as in the London and Paris basins, the remains of quadrupeds are found. The type of this era is greatly advanced, and animals of a more perfect organization are seen to have flourished. These tertiary beds present a set of animals almost entirely new, "and as we ascend in the series, we find more and more of these identical with species still existing upon earth, as if we had now reached the dawn of the present state of the zoology of our planet."

Cuvier ascertained about fifty species of mammifers in the Paris basin, and others have been discovered since his time. These are all extinct, but they belonged to the highest class of animals, and are represented by the horse, camel, fox, wolf, tiger, &c., of our day.

This brings us up to the carnivora, "a considerably elevated point in the animal scale, but still leaving a blank for the quadrumana (monkeys) and for man."

With the superficial formations we have the commencement of the present species of animals. In various caves the osseous remains of the existing families of common quadrupeds and birds have been found in great abundance. Many of the bones of the gentler sort of animals are broken, and it is hence inferred that the caves were the haunt of hyenas and other predaceous animals, by which the smaller ones were consumed.

All this has the air of truthful history. The record is written in characters which appear to admit of no question. In this manner creation would seem to have progressed, from simple to the higher forms of organization.

"Amongst plants, we have first sea weeds, afterwards land plants; and amongst these the simpler before the more complex. In the department of zoology, we see, first, traces all but certain of infusoria; then polypiaria, crinoidea, and some humble forms of the mollusca; afterwards higher forms of the mollusca; and it appears that these existed for ages before there were any higher types of being. The first step forward gives fishes, the humblest class of the vertebrata; and, moreover, the earliest fishes partake of the character of the lower sub-kingdom, the articulata. Afterwards come land animals, of which the first are reptiles, universally allowed to be the type next in advance from fishes, and to be connected with these by links of insensible gradation. From reptiles we advance to birds, and thence to mammalia, which are commenced by marsupialia, acknowledgedly low forms of their class. That there is thus a progress of some kind the most superficial glance at the geological history is sufficient to convince us." p. 110.

This progress of organic life appears to have observed some correspondence with the progress of physical conditions on the surface of the earth. In other words, when the globe attained the fit state to support particular tribes of animals, they made their appearance upon it. The huge saurians were adapted to the low muddy coasts and sea-margins of the time when they flourished. Marsupials appear at the time when the surface was in that state in which we find Australia, where they now live in the greatest abundance, namely flat, and imperfectly variegated. And when the land and sea had come into their present relations, man with the animals that now surround him, was placed upon the earth. The land animals could hardly have existed before the carbonigenous era, "owing to the great charge of carbonic acid gas presumed to have been contained in the atmosphere down to that time."

And this introduces our author's "considerations on the origin of the animated tribes." That God created them, "as well as the terraqueous theatre of their being, is a fact so powerfully evidenced, and so universally received," that he at once takes it for granted. But in the particulars of this "so highly supported idea," he thinks there is "cause for some reconsideration." He is not willing to receive the scriptural account of the matter. He cannot believe that every shellfish and reptile was a special creation. This idea appears to him ridiculous. Much more worthy of the Creator he holds the supposition, that all things have been progressively developed, the higher out of the lower, by the intervention of law. He adopts the doctrine of spontaneous generation. The waters having reached a stage adapted to fish, brought forth the finny tribes; the earth, in due season, shot up the oak and the pine; and tigers and hyenas came forth of some of the humbler tribes of the mammalia. Monkeys, after a succession of ages, were developed out of the highest of the mammifers, and they, the period of utero-gestation having been, by some means, lengthened, brought forth infants that grew to be men. This is the theory of creation which this writer proposes to substitute for the one upon which the christian mind has so long rested. "Unquestionably," he says, "what we ordinarily see of nature is calculated to impress a conviction that each species invariably produces its like;" which is equivalent to saying, that all that we ordinarily see of nature stamps absurdity upon his doctrine. But he finds in Mr. Babbages "calculating machine', what he fancies to be "a remarkable illustration of natural law." This machine is moved by a weight, "and presents to the eye of the beholder successively a series of numbers engraved on its divided circumference." It continues for a long time to produce the same series of natural numbers. After seeing five hundred turns most persons would believe that it would go on regularly forever, and after the fifty thousandth turn, perhaps no one would doubt that the next turn would be fifty thousand and one. So it will be; and the machine continues to turn up the expected number from *one* up to *one hundred million.* "True to the vast induction which has been made, the next succeeding turn will be one hundred million and one; but the next number presented by the rim of the wheel, instead of being one hundred million and two, is one hundred million *ten thousand* and two."*

Here we have the doctrine fully, in this illustration. The monkeys went on giving birth to quadrumana for, it may be, one hundred million of generations and one, and then, instead of young monkeys, the strange progeny were "unplumed, risible bipeds"—"cooking animals"—the first of the genus *homo.* And this is the hypothesis which the respectable writer in the Medico-Chirurgical Review says "is three or four centuries in advance of the times." Then, indeed, are we "*advancing backwards*," for the hypothesis is as old, at least, as the time of Lord Monboddo, whose name, on account of its association with this ridiculous conjecture, has been a name to be laughed at for almost a century.

There are two objections, either of which would be fatal

* Babbage in the 9th Bridgewater Treatise.

to this hypothesis. One is, that in all the six thousand years during which the human race has inhabited the earth, nothing has been witnessed that gives the slightest countenance to it. The author admits that this is the fact, but he meets the objection by saying that this testimony is negative merely. The present era has lasted some thousands of years, but who can say that it was not introduced in the way imagined, and may not issue in some such birth as this law of development requires? Things, he contends, may proceed for many generations yet as they have done since the beginning of the species, and then a new and superior race of beings spring from the present imperfect family of man. But the answer is not satisfactory; the negative proof must be held to be good if not met by any of an opposite character. Men will believe in the stability of nature so long as they see her processes going forward with unerring exactitude; they cannot help believing that parturitions, which have gone on without any approach to such a deviation as this hypothesis supposes, for eighteen thousand generations, had a specific, determinate beginning, and will go on without essential change while the race endures. No analogy of a "Babbage calculating machine" will overturn or shake this belief. In strength it approaches to the nature of an instinctive faith. That which men sow, they expect, with an unwavering confidence, to reap. They do not look for grapes on thorns, or figs on thistles. From the horse they are sure of deriving a race of horses, and from their cows, cattle of the same kind. They are never disappointed by finding a calf where it should have been a colt, or a puppy instead of a pig. If there is any exception to this law it is to be found in the Kentucky boatman, who boasts of being "*half horse and half alligator, with a little touch of the snapping turtle;*" but, for ourselves, we are not prepared to surrender the law upon the strength of this exception. Nature does not more abhor a vacuum than such monstrous births.

[TO BE CONTINUED.]

